# RETRACTION: Evaluating the Results of Eyebrow Lift by Combining Methods of Subcutaneous Flap and Thread Support in Patients with Droopy Eyebrows

**DOI:** 10.1111/srt.70210

**Published:** 2025-07-21

**RETRACTION**: G. Shirani, R. Jalilian, E. Shafiei, F. Mohammadi, A. Shamshiri, and H. Lotfi, “Evaluating the Results of Eyebrow Lift by Combining Methods of Subcutaneous Flap and Thread Support in Patients With Droopy Eyebrows,” *Skin Research and Technology* 29, no. 2 (2023): e13284, https://doi.org/10.1111/srt.13284.

The above article, published online on 14 February 2023 in Wiley Online Library (wileyonlinelibrary.com), has been retracted by agreement between *Skin Research and Technology*; and John Wiley & Sons Ltd. The article has been accepted for publication following an inadequate peer review process. Following publication of the article, several flaws and inconsistencies were identified including, but not limited to, the reported statistical analyses and duplication of images between Figures 2B and 2C. Additionally, although the study involved the application of a novel procedure on human subjects, no statement of ethical approval is provided. In light of these issues, the article has been retracted. The authors have been informed of this decision but unavailable for a final confirmation.

